# Efficient Quenching of Two-Photon Absorption Induced Photoluminescence in Carbon Nanodots for Fe^3+^ Ion Detection

**DOI:** 10.1021/acsomega.5c01915

**Published:** 2025-06-26

**Authors:** Agnieszka Siomra, Dominika Wawrzyńczyk, Bartłomiej Cichy, Magdalena Wądrzyk, Paulina Kasperkiewicz, Marek Samoć, Marcin Nyk

**Affiliations:** † Institute of Advanced Materials, Faculty of Chemistry, 214839 Wroclaw University of Science and Technology, Wybrzeze Wyspianskiego 27, 50-370 Wroclaw, Poland; ‡ Polish Academy of Sciences, 49567 Institute of Low Temperature and Structure Research, Okolna 2, 50-422 Wroclaw, Poland; § Department of Chemical Biology and Bioimaging, Wroclaw University of Science and Technology, Na Grobli 15, 50-421 Wroclaw, Poland

## Abstract

This study reports on the linear and nonlinear optical (NLO) properties of water-dispersed carbon nanodots (CNDs) fabricated via a rapid one-step hydrothermal microwave-assisted technique. The CNDs exhibit two-photon excited luminescence, which was characterized with spectrally tunable femtosecond laser pulses as involving the two-photon absorption (TPA) cross sections (σ_2_) as large as 1.4 × 10^3^ Goeppert-Mayer (GM) at the excitation wavelength of 720 nm and the quantum yield (QYs) of 28%. By analyzing the σ_2_ spectra, specific wavelength ranges optimal for excitation via the two-photon process were identified. In addition, the potential of the CNDs as sensors for the selective and sensitive detection of Fe^3+^ ions through one- and two-photon induced fluorescence quenching was investigated. To gain deeper insights into the mechanism underlying the observed decrease in fluorescence intensity upon addition of Fe^3+^ ions, potentially involving dynamic quenching, fluorescence quenching experiments across various temperatures were conducted, being the first such study in both one-photon and two-photon excitation regimes for this sensor. The possibility of energy transfer between CNDs and Fe^3+^ ions was investigated by analyzing the luminescence kinetics using time-correlated single-photon counting (TCSPC) and streak camera techniques, in one- and two-photon regime, respectively. The pronounced nonlinear optical response of the CNDs highlights their potential as active optoelectronic materials for optical sensors operating in the near-infrared (NIR) region. Cytotoxicity studies of the water-dispersed CNDs revealed no observable toxicity, even at high concentrations, making them suitable for biorelated applications.

## Graphical abstract

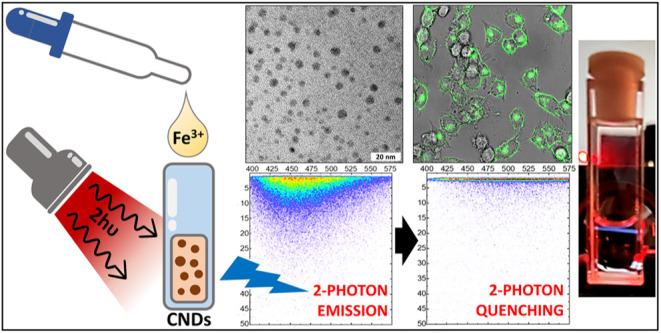

## Introduction

Over the years, carbon-based nanomaterials have proven to be a key class of materials in nanotechnology, offering unparalleled versatility and functionality that enable a wide range of properties and applications. Among them, carbon nanodots (CNDs), discovered accidentally by Xu et al.,^1^ have attracted significant interest due to their remarkable physical and chemical properties, including tunable photoluminescence (PL), excellent photostability, low cytotoxicity, good water dispersibility, and readily achievable surface modification. Moreover, CNDs offer several advantages in comparison to other fluorescent nanomaterials or organic molecules. For instance, many conventional organic fluorescent dyes have high fluorescence quantum yields (QYs) but are difficult to synthesize and exhibit poor photostability in aqueous conditions.^2–4^ CNDs often outperform semiconductor quantum dots (QDs) and are a preferred choice for biorelated applications as they do not include toxic heavy metals.^5^ Furthermore, CNDs can also surpass metal nanoclusters in terms of stability and QYs values, making them an attractive metal-free alternative.^6^ The facile synthesis, surface functionalization, and doping methods offer a broad range of possibilities for tailoring the CNDs properties to suit specific applications and position them as ideal candidates for use in e.g., optoelectronics, photovoltaics, catalysis, bioimaging, and analytical sensing.

To date, a wide range of techniques has been employed for the detection of metal ions. While methods such as atomic absorption spectroscopy (AAS) and inductively coupled plasma mass spectrometry (ICP-MS) provide excellent sensitivity and selectivity, they often entail operational complexities and lengthy sample preparation and analysis times. Furthermore, many traditional methods are predominantly laboratory-bound, rendering them unsuitable for on-site detection. In contrast, fluorescent sensors based on nanomaterials offer several practical advantages over these conventional techniques, as they excel in areas such as simplicity, cost-effectiveness, and rapid real-time applications and may be compared to other user-friendly and low-cost methods, including electrochemical and colorimetric techniques.^7^ Since the various nanoparticles can be easily functionalized and their optical properties can be readily tuned, they are quite flexible in adapting to various detection targets and purposes. Interestingly, the CNDs inherently function as fluorescent sensors for a variety of metal ions, with their sensitivity and selectivity being closely tied to the nature of the carbon source. For example, in 2012, Lu et al. synthesized fluorescent CNDs from sweet potatoes and demonstrated their potential for selective Hg^2+^ ions detection.^8^ A year later, Mohd Yazid et al. reported CNDs derived from sago starch nanoparticles as an optical probe for Sn^2+^ ions.^9^ In 2017, Shi et al. reported CNDs synthesized using 4-amino salicylic acid for Al^3+^ ions sensing,^10^ while Wu et al. implemented a fluorescent sensor array based on different types of CNDs that allowed the detection and differentiation of Ag^+^, Cd^2+^, Cr^2+^, Fe^3+^, Hg^2+^, and Pb^2+^ ions.^11^ Numerous reports have concentrated on utilizing CNDs for the fluorescence-based detection of Fe^3+^ ions, simultaneously highlighting their potential for sensing in biological systems.^12–15^ The performance of CNDs as sensors for metal ion detection is usually investigated only in the one-photon regime, under UV excitation. The two-photon regime, where the excitation proceeds by the simultaneous absorption of two low-energy photons, holds significant importance in the context of biological and medical applications. Irradiation with near-infrared (NIR) photons using femtosecond lasers is safer for tissues and cells since the use of low-energy photons and the spatial localization of the excitation at the focus of the excitation beam minimize the risk of photobleaching and photodamage. A NIR beam can also penetrate deeper into tissues compared with the visible or UV light used in single-photon excitation. Following the key studies investigating the behavior of the two-photon induced emission of CNDs,^16,17^ several authors have demonstrated the usefulness of CNDs-based sensors in the two-photon regime; however, the reported nanodots were either functionalized with organic molecules or the explored spectral range of two-photon excitation wavelengths was relatively narrow.^18–22^

In this work, we explore in a quantitative manner the nonlinear optical behavior of a label-free fluorescent sensor for Fe^3+^ ion detection based on CNDs derived from urea and citric acid in a broad spectral range of wavelengths (∼600 to 1000 nm), which allows for accurate optimization of the two-photon excitation wavelength for enhanced sensing activity of the synthesized nanodots. The primary objective of this paper is to investigate the observed fluorescence quenching mechanism through temperature-dependent measurements conducted not only in the single-photon but also in the two-photon regime, an aspect that, to the best of our knowledge, has not yet been reported for CND-based sensors for Fe^3+^ detection.

## Results and Discussion

### Morphological Analysis and Structural Characterization

As shown in Figure 1A, the synthesized CNDs were not aggregated and exhibited a spherical shape, with sizes varying from 3.10 to 7.55 nm. The mean diameter of the nanodots was found to be 5.12 ± 0.25 nm (see: inset of Figure 1A). The energy-dispersive X-ray (EDX) analysis with spectrum displayed in Figure 1B revealed the presence of carbon, oxygen, and nitrogen in the sample, confirming the successful synthesis of nitrogen rich CNDs. The peaks corresponding to copper originate from the grid used during the measurements. Diffused rings observed in the selected area electron diffraction (SAED) patterns of the synthesized CNDs (see: inset of Figure 1B) suggest the presence of an amorphous phase of carbon. The lack of long-range structural order of the investigated CNDs was further confirmed by the powder X-ray diffraction (PXRD) measurements, which revealed a broad peak at 2θ = 24°, attributed to the (002) plane of the graphitic framework with a *d*-spacing of approximately 0.37 nm (Figure S1). In crystalline graphite, this value is equal to 0.34 nm and the increase in lattice spacing in CNDs might result from the presence of oxygen-bearing functional groups.^23^

**Figure 1:**
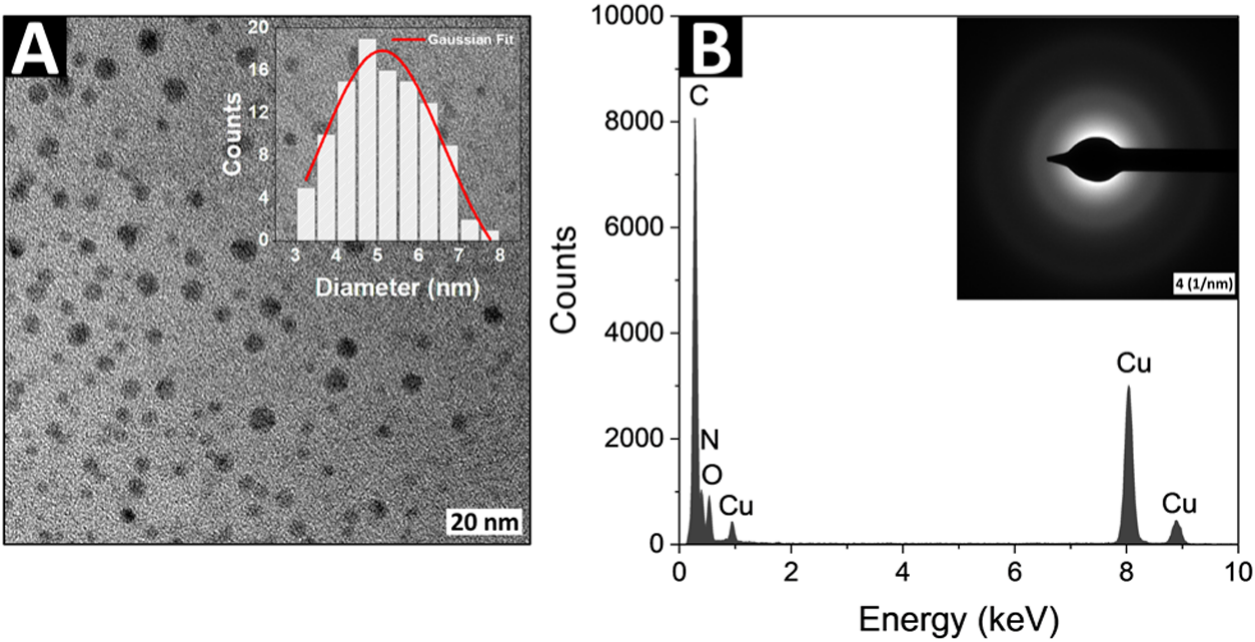
(A) Representative TEM image and its size distribution histogram (inset) and (B) the EDX spectra and SAED (inset) of the synthesized CNDs.

### Optical Properties

The optical properties of the colloidal CNDs were characterized by room temperature UV–vis absorption and luminescence emission spectra (Figure 2A). The narrow band with a maximum at 335 nm, observed in the absorption spectrum, is typical for absorption characteristics of colloidal CNDs and corresponds to the n−π* transition of C=O bonds.^24–26^ The CNDs exhibit strong luminescence, both their intensity and peak wavelength being dependent on the excitation wavelength. Notably, the excitation wavelength of 360 nm resulted in the highest emission intensity (Figure S2). Figure 2B illustrates the red-shift of the emission with increasing excitation wavelength, consistent with findings reported in previous studies.^27,28^ It is to be noted that the red-shift is more prominent above the excitation wavelength threshold of 360 nm, which corresponds well with results obtained by Siddique et al., who proposed that electronic transitions mediated by O-related surface states and edge states account for excitation-dependent photoluminescence above the threshold wavelength.^29^ The experiment shown in the photograph presented in Figure 2C illustrates intense visible emission obtained under CW laser diode excitation at 360 nm.

**Figure 2:**
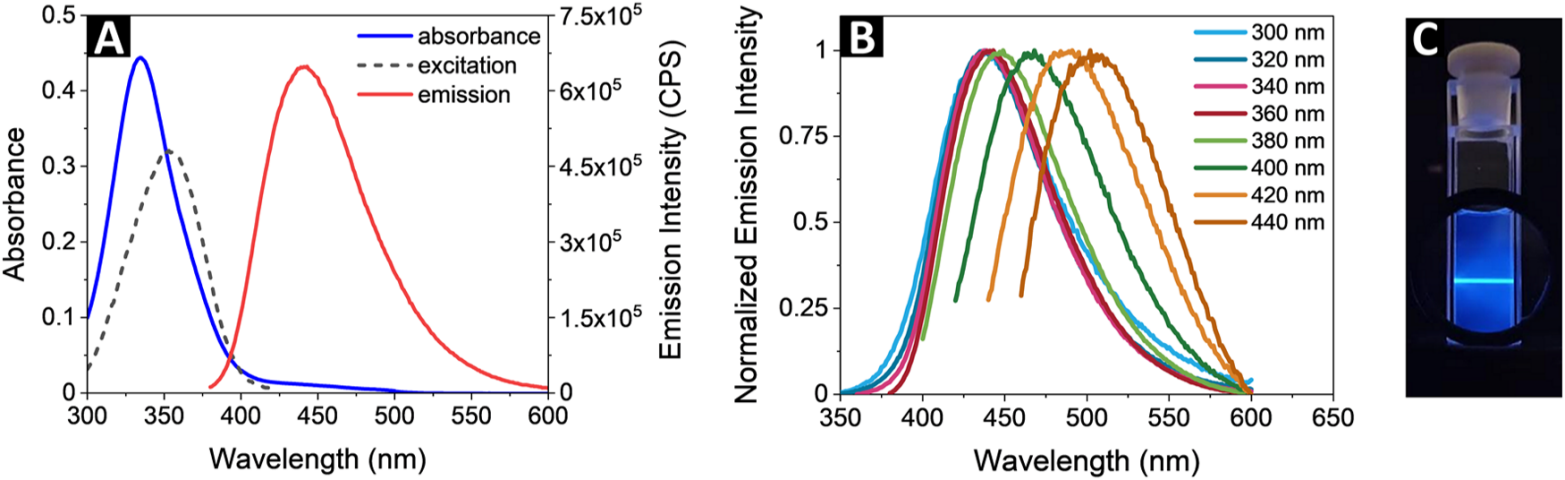
(A) UV–vis absorbance, excitation and emission (λ_exc._ = 360 nm) spectra of the aqueous solution of CNDs, (B) normalized fluorescence intensity of the aqueous solution of CNDs at different excitation wavelengths, and (C) an experiment illustrating efficient visible luminescence under excitation with a CW laser diode at 360 nm (400 nm long-pass filter inserted to reduce the scattering).

To investigate the nonlinear optical (NLO) behavior of the CNDs, the two-photon excited emission (TPEE) technique was employed and a broad range of excitation wavelengths from 640 to 960 nm was explored. Using the TPEE method, as summarized by Makarov et al.,^30^ the values of two-photon absorption (TPA) cross sections, σ_2_, based on comparison of the emissive properties of the studied CNDs and the reference dye, were determined (see details in Supporting Information). The representative two-photon induced emission spectra of the CNDs at different excitation wavelengths are shown in Figure 3A. The maximum TPA cross-section value of 1422 GM (Goeppert-Mayer units, where 1 GM = 10^–50^ cm^4^ s per photon) was actually found at that excitation wavelength, indicating that the nonlinear absorption is the strongest at a wavelength approximately twice that of the maximum of the one-photon absorption (OPA) band of the CNDs (Figure 3B). The broad shoulder between 780 and 900 nm in the TPA cross-section plot, illustrated in Figure 3B, aligns closely with the less significant TPA cross sections band of the CNDs, previously reported by Barhum et al.^20^ This correlation suggests that the enhanced TPA characteristics in this region are consistent with the behavior seen in similar carbon dots systems. To verify the order of the multiphoton excitation process, the dependence of the integrated photoluminescence intensity on the excitation laser beam power at the wavelength corresponding to the maximum value of σ_2_ was measured. Figure 3C presents this dependence on a log–log scale. The experimental data are well fitted by a straight line with a slope of ∼2.1, confirming that under 720 nm femtosecond laser excitation, the observed process is a two-photon absorption. Figure 3D depicts an experiment in which a microscope objective lens focused a beam at 720 nm from a femtosecond pulsed laser system into a cuvette with the CNDs. The resulting emission, clearly visible to the naked eye as a bright spot, is concentrated in a highly localized volume at the focus, demonstrating a superlinear dependence on the excitation light intensity. Such photographs are usually presented as proof of the intrinsic capability of the two-photon absorption process to be used for three-dimensional optical imaging.

**Figure 3:**
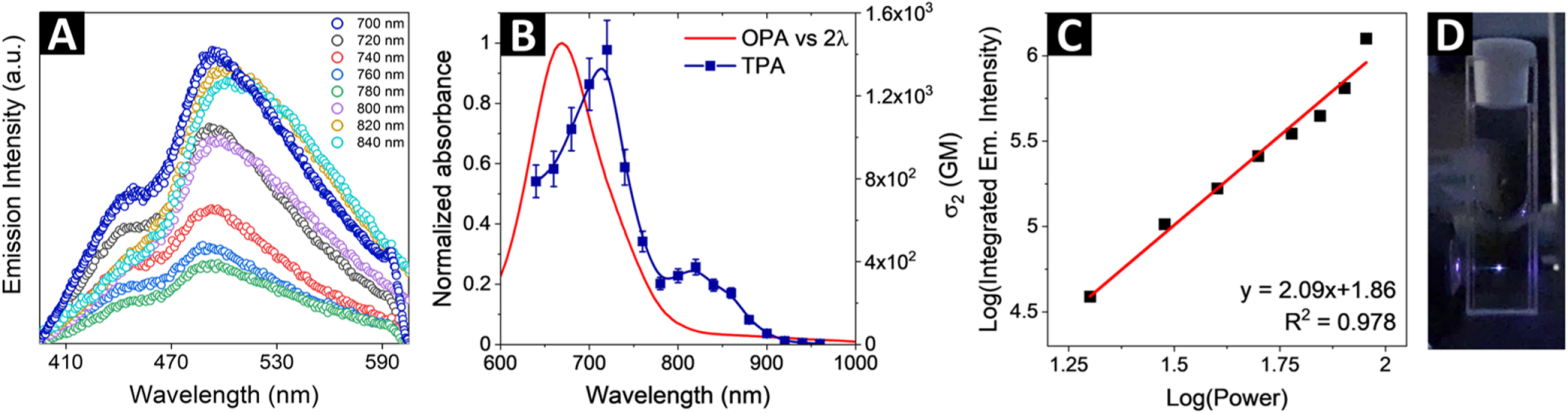
(A) Representative two-photon induced emission spectra of CNDs at different excitation wavelengths, (B) wavelength dependence of the measured and calculated TPA cross sections of the CNDs (blue line drawn to guide the eyes) versus the absorbance spectra under one-photon excitation plotted against 2λ, (C) log–log plot presenting the integrated two-photon induced emission intensity vs power of the incident laser beam, (D) an experiment illustrating efficient two-photon induced photoluminescence spot under femtosecond pulsed laser operating at 720 nm.

For the evaluation of the relative merits of the emerging two-photon active materials, the TPA cross section (σ_2_) as well as the two-photon action cross section (also referred to as two-photon brightness, σ_2_ × QY), can be scaled in regard to molecular weight (MW) of a single nanoparticle, estimated using the size of nanoparticle and the density of the bulk material, to give the σ_2_/MW and (σ_2_ × QY)/MW parameters, respectively. These merit factors provide a useful comparison between disparate types of two-photon absorbers, particularly in the evaluation of nanosystems of varying composition or sizes. In the case of the studied CNDs, the σ_2_/MW highest value was found to be 1.6 × 10^–2^ GM mol g^–1^, while the (σ_2_ × QY)/MW factor was calculated at 4.6 × 10^–2^ GM mol g^–1^ at 720 nm.

The peak values of the TPA cross sections of the CNDs determined through TPEE experiments were further compared to those of other emerging two-photon active materials and are summarized in Table 1. The CNDs presented in this study demonstrate σ_2_ and σ_2_ × QY values that are comparable to or, in some cases, higher than those reported for other carbon dots and various cadmium-free nanomaterials, such as InP@ZnS and SQDs, as well as some organic fluorescent probes (see: Table 1). It is worth noting that literature cites CNDs with exceptionally high two-photon cross-section values, some reaching up to ∼50 × 10^3^ GM.^21,38,39^ However, while the studied CNDs may demonstrate lower σ_2_ values in comparison to these exceptional cases, they maintain an advantageous balance between rapid, efficient synthesis and desirable optical properties; the above-mentioned materials typically require several hours for synthesis, whereas the CNDs in this study can be prepared in only 10 min.

**Table 1:** Maximum Values of the Two-Photon Absorption Cross Sections (σ_2_), Molecular Weight Normalized Two-Photon Absorption Cross Sections (σ_2_/MW), Quantum Yields (QY), Two-Photon Action Cross Sections (σ_2_ × QY) and Normalized Two-Photon Action Cross Sections (σ_2_ × QY/MW) of Selected Cadmium-Free Nanoparticles and Organic Fluorescent Probes, Including Coumarin 153 as a Reference Dye for TPEE Method

material (size)	solvent	technique	λ_exc._ [nm]	σ_2_ [GM]	σ_2_/MW [GM·mol/g]	QY [%]	σ_2_·QY [GM]	σ_2_·QY/MW [GM·mol/g]	references
carbon nanodots (∼5 nm)	water	TPEE	720	1.4 × 10^3^	1.6 × 10^–2^	28	4 × 10^2^	4.6 × 10^–3^	this work
phenylenediamine CDs (∼3 to 5 nm)	ethanol	Z-scan	830	∼50	1.2 × 10^–3^ ^a^	35	0.2 × 10^2^	4.3 × 10^–3^ ^a^	^20^
core–shell InP@ZnS QDs (∼4.3 nm)	toluene	Z-scan	880	2.2 × 10^3^	18 × 10^–3^	31	6.8 × 10^2^	5.5 × 10^–3^	^31^
SQDs from NaOH precursor (∼5.6 nm)	water	TPEE	780	1.85 × 10^2^	1.8 × 10^–3^	0.8	1.5	1.4 × 10^–5^	^32^
carbonized polymer dots (∼2.7 nm)	water	TPEE	775	∼20	1.5 × 10^–3^ ^a^	14.3	2.9	2.2 × 10^–4^ ^a^	^33^
CDs from PAB precursor (∼4 nm)	ethanol	TPEE	700	1.5 × 10^3^	3.4 × 10^–2^ ^a^	18.2	2.6 × 10^2^	6.1 × 10^–3^ ^a^	^34^
DMI	saturated RNA Tris–HCl	TPEE	760	981	2.3	2.2	22.1	5.3 × 10^–2^	^35^
difluoroborate dyes	chloroform	Z-scan	925	280–404		2–46	6–1.7 × 10^2^		^36^
DPND-based dye	dichloromethane	Z-scan	820	5.2 × 10^3^	9.3	1.6	83	14.9 × 10^–2^	^37^
Coumarin 153	carbon tetrachloride	TPEE	800	45	0.15	64	28.8	9.3 × 10^–2^	^30^, this work

a Values estimated based on the given nanoparticle size and the density of the bulk material data obtained from existing literature.

### Optical Metal Ions Sensing

In the first step, the fluorescence response of the colloidal aqueous CNDs was investigated in the one-photon regime in the presence of five selected ions: Cu^2+^, Cd^2+^, Al^3+^, Cr^3+^, and Fe^3+^, many of them being among the most commonly found heavy metals in wastewater.^40^ The intensities of the emission spectra of the studied CNDs upon addition of metal ions did not show strong changes, except for the case of Fe^3+^ where significant quenching was observed (Figure S3).

This effect is most likely the result of high affinity of ferric ions to functional groups carrying oxygen and nitrogen on the surface of the CNDs in comparison with other cations.^41^ The emission response upon addition of ferric ions solution under 360 nm excitation is shown in Figure 4A. The selectivity toward Fe^3+^ in the presence of different amounts of interferents (here: Cu^2+^, Cd^2+^, Al^3+^, and Cr^3+^) was also confirmed (Figure S4). The quenching of the luminescence by Fe^3+^ ions was further interpreted in terms of the Stern–Volmer (SV) equation^42^

1 $$\frac{F_{0}}{F}=1+K_{\mathrm{SV}}\left[Q\right]$$

where *F*_0_ and *F* are the integrated fluorescence emission intensities of aqueous solution of CNDs in the absence and in the presence of ferric ions, respectively, *Q* is the quencher concentration (ferric ions), and *K*_sv_ denotes the Stern–Volmer constant. It was found that for low concentrations of ferric ions (0–0.25 mM), the SV plot deviates from linearity and the dependence is better described by an exponential function (Figure 4B). The limit of detection (LoD) and limit of quantification (LoQ) for the studied CNDs were estimated using formulas

2 $$\mathrm{LoD}=\frac{3.3s}{K_{\mathrm{SV}}}$$

3 $$\mathrm{LoQ}=\frac{10s}{K_{\mathrm{SV}}}$$

where *s* denotes the standard deviation of the response, and were found to be 0.053 mM (53 μM) and 0.160 mM (160 μM), respectively. As shown in Figure 4C, the plot of the *F*_0_/*F* value against the concentration of ferric ions exhibits a linear relationship over the quencher concentration range of 0.25–3.5 mM, with an *R* ^2^ value of 0.991.

**Figure 4:**
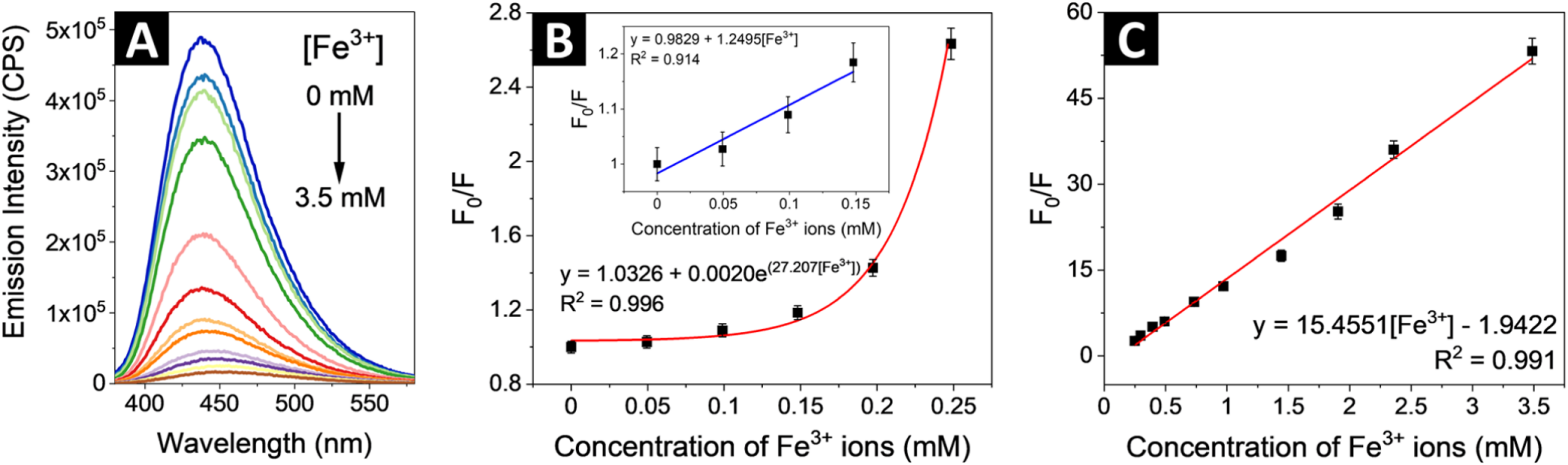
(A) Single-photon excited emission spectra of CNDs solution (λ_exc._ = 360 nm), demonstrating the fluorescence quenching upon increasing concentration of Fe^3+^ ions, (B) Stern–Volmer plot in low concentration range (inset: linear relationship used to estimate LoD and LoQ) and (C) Stern–Volmer plot in high concentration range of ferric ions. The intensity of fluorescence was measured at the excitation wavelength of 360 nm after the incubation time of 1.5 min.

The activity of the obtained CNDs as a potential Fe^3+^ ions sensor was also investigated in the two-photon regime, under femtosecond laser excitation at the wavelength of 720 nm, which is the wavelength where the nonlinear absorption was found to be the strongest (see: Figure 3B). The representative two-photon induced emission spectra recorded upon addition of ferric ions to the CNDs solution are presented in Figure 5A. Similar to the results obtained in the one-photon regime, the SV plot deviates from the linearity of over the range of low concentrations of ferric ions, where the relationship between *F*_0_/*F* and the quencher concentration can be described by an exponential function, and a good linear fit with an *R* ^2^ value of 0.953 is obtained in the range of 0.25–8.25 mM (Figure 5B,C). The estimated LoD and LoQ for the studied CNDs under two-photon excitation were found to be 0.080 mM (80 μM) and 0.256 mM (256 μM), respectively. In both cases, a linear SV plot is obtained in the range of high concentrations of ferric ions (>0.25 mM).

**Figure 5:**
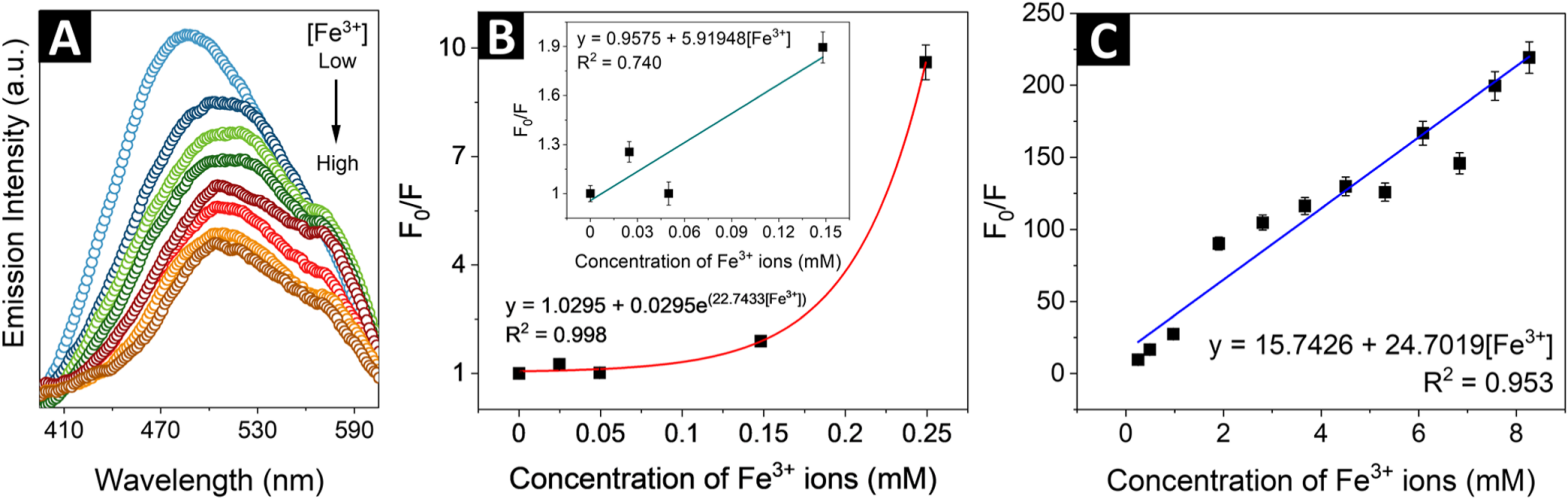
(A) Representative two-photon excited emission spectra of CNDs solution (λ_exc._ = 720 nm), demonstrating the fluorescence quenching upon increasing concentration of Fe^3+^ ions, (B) Stern–Volmer plot for CNDs in the low concentration range (inset: linear relationship used to estimate LoD and LoQ), and (C) Stern–Volmer plot in the high concentration range of ferric ions. The intensity of fluorescence was measured at the excitation wavelength of 720 nm after the incubation time of 1.5 min.

While recent advancements in ferric ion sensors primarily target the detection of trace amounts (see: Table S1), sensors optimized for detecting higher concentrations are also valuable, being relevant in industrial and environmental contexts, where metal ion levels are inherently higher and there is a need of measuring or tracking large-scale concentrations of these ions. These applications demand robust sensor performance in environments with elevated ferric ion concentrations, where extreme sensitivity is not a critical factor. Additionally, it is crucial to characterize the behavior of sensors at higher concentrations of analyte, as models that accurately describe sensor performance at low concentrations often do not extrapolate well to the high concentrations range.

The positive deviation from linearity of the SV plot (an upward curve) observed at low concentrations of ferric ions in both one-photon (λ_exc._ = 360 nm) and two-photon (λ_exc._ = 720 nm) regimes might suggest that both static and dynamic quenching contribute to the observed decrease in fluorescence intensities. The classical Stern–Volmer model does not allow to distinguish between dynamic and static quenching or to determine whether the decrease in emission intensity of the studied system is resulting from one or two mechanisms.^43^ To investigate the mechanism behind the observed quenching of CNDs photoluminescence, fluorescence lifetime measurements and temperature-based quenching measurements were conducted.

### Fluorescence Quenching Mechanism

The effects of the addition of Fe^3+^ ions on fluorescence lifetimes of the CNDs were measured using the time-correlated single-photon counting (TCSPC) technique. The fluorescence decay curves were fitted with a double-exponential model, and the obtained values of the short and long lifetime decay components, along with their respective contributions to the mean lifetime, are summarized in Table S2. The decrease in mean photoluminescence (PL) lifetime upon the addition of ferric ions is shown in Figure 6A, while the representative decay curves (for as-synthesized CNDs and after consecutive addition of 100 and 200 μL of Fe^3+^ solution) together with fitting lines are presented in Figure 6B. Based on the decay curves, it is clear that the luminescence lifetime is reduced upon addition of Fe^3+^ into the CNDs solution. The TCSPC data were also analyzed using the slope of the SV plot. There is a clear linear dependence (*R* ^2^ = 0.946) between the τ_0_/τ parameter, where τ_0_ represents the mean PL lifetime of the as-synthesized CNDs and τ represents the mean PL lifetime of CNDs after the addition of the Fe^3+^ solution, and the concentration of the quencher (see: inset of Figure 6A), indicating that the decay rate accelerates as the Fe^3+^ concentration increases.

**Figure 6:**
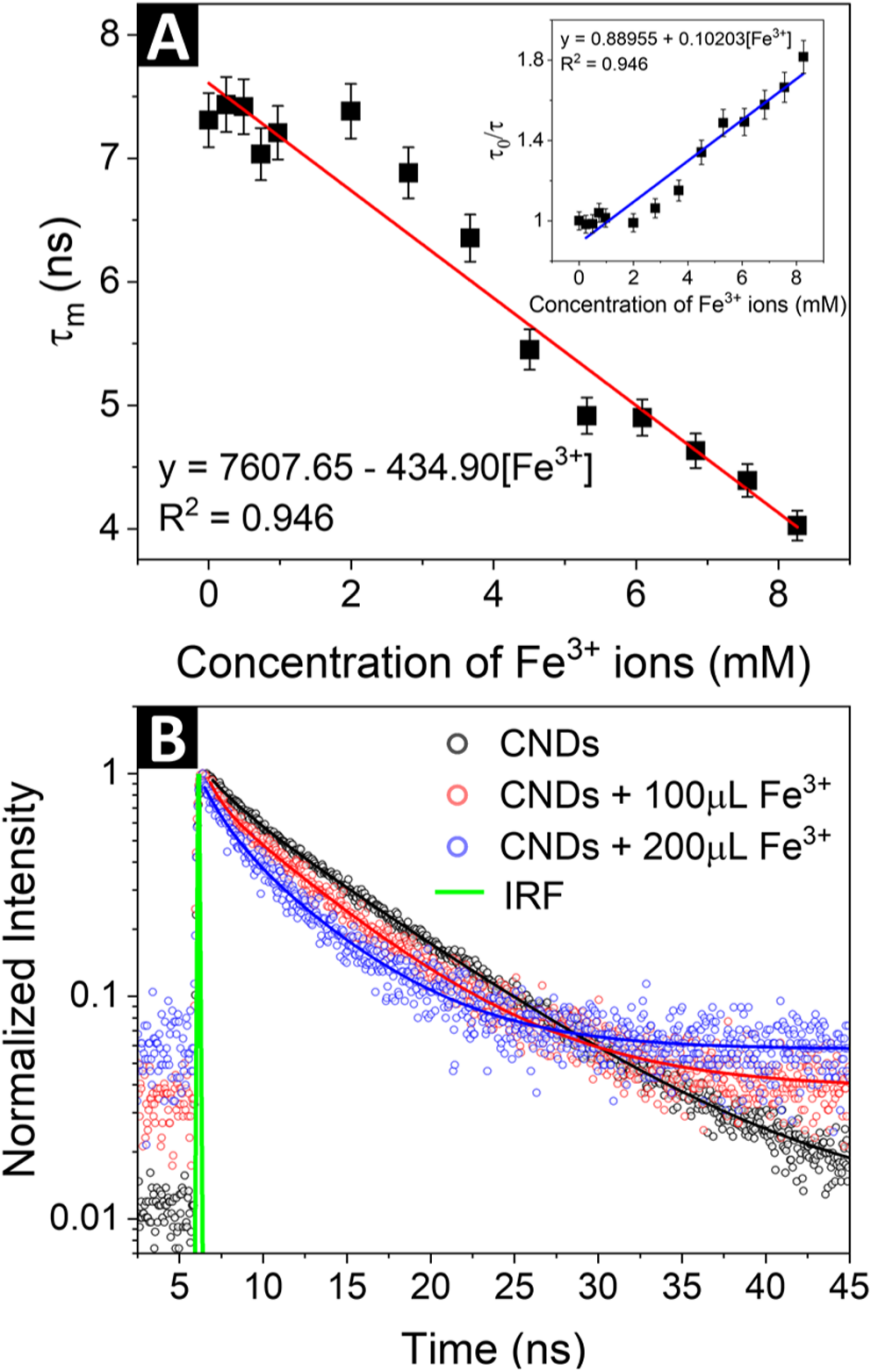
(A) Calculated mean PL lifetimes of CNDs as a function of Fe^3+^ ion solution with fitting showing a linear trend (inset: Stern–Volmer plot of the fluorescence lifetimes upon addition of ferric ions) and (B) representative decay curves for as-synthesized CNDs and after consecutive addition of 100 and 200 μL of Fe^3+^ ions solution with fitting lines and instrument response function (IRF) for comparison.

Corresponding experiments on luminescence quenching were also conducted for the two-photon excitation regime, i.e., λ_exc._ = 720 nm. The fluorescence intensity time-resolved maps were captured with a streak camera (Figure 7) and the corresponding τ values were calculated based on single-exponential fitting of luminescence traces (Table S3). Similar to the case of one-photon excitation, the nonlinear regime showed potential for Fe^3+^ sensing based on the decay kinetics measurements. When comparing the time-resolved photoluminescence spectra of as-synthesized CNDs (Figure 7A) to the corresponding ones measured after addition of consecutive amounts of Fe^3+^ solution (Figure 7B–F), dramatic changes in two-photon excited emission kinetics are observed even after addition of 5 μL of Fe^3+^ solution (Figure 7B). The values of τ were reduced from 6.6 ns for as-synthesized CNDs, down to ∼300 ps for 40 μL of Fe^3+^ solution added (Table S3). The presence of the decrease of fluorescence intensity and lifetime reduction is, however, insufficient to differentiate between energy transfer and electron transfer, as both processes can lead to these effects. Therefore, to clarify the underlying quenching mechanism, both in the linear and nonlinear optical regimes, temperature-dependent measurements were performed.

**Figure 7:**
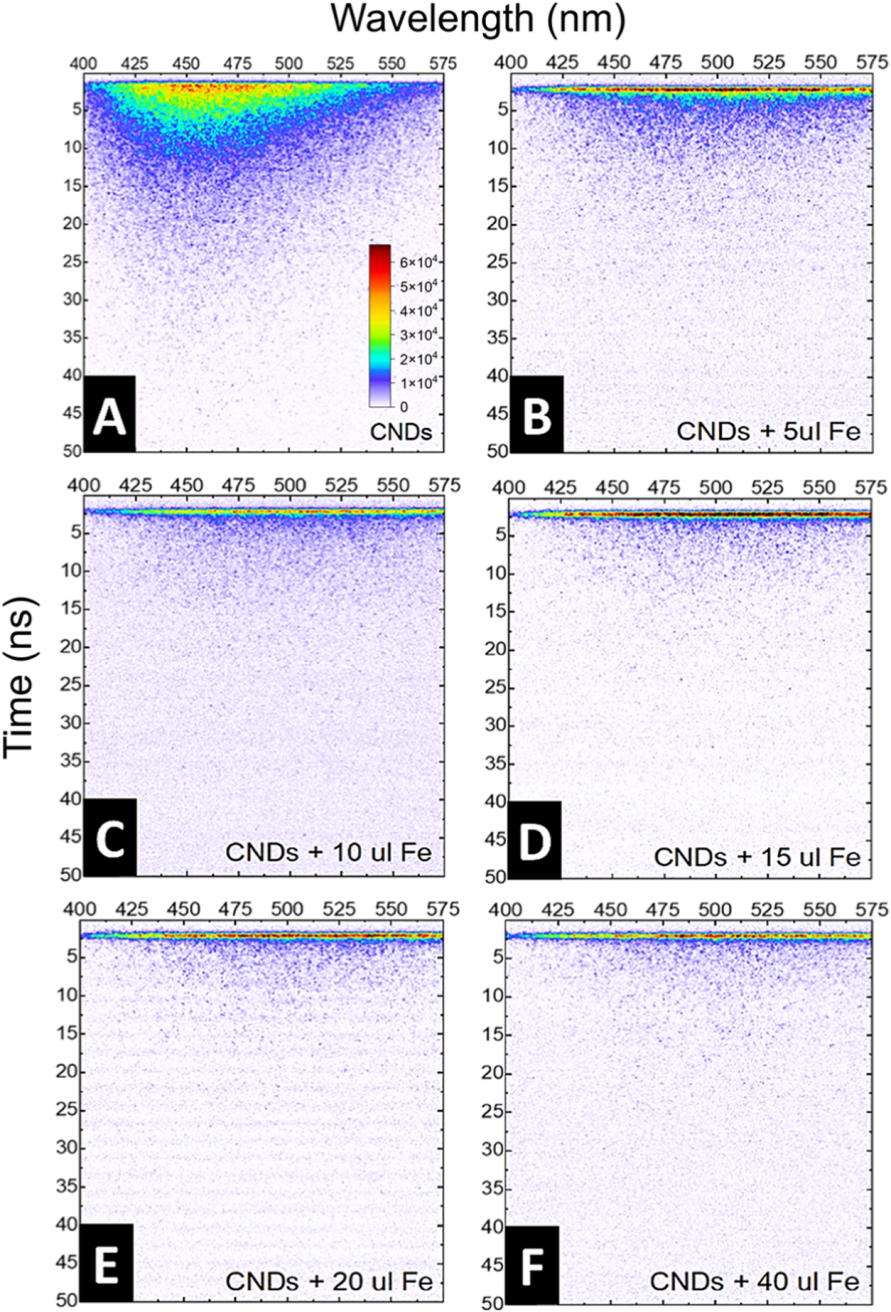
(A) Time-resolved photoluminescence spectra of the (A) as-synthesized CNDs and (B–F) after addition of consecutive amounts of Fe^3+^ ions solution.

The measurements covering the range from 15 to 45 °C were performed in a Peltier-controlled cuvette holder under both one- and two-photon excitation. The results are presented in Table 2. With the increase of the temperature, the slope of the SV plot increases over the range of quencher concentrations from 0 to 1.2 mM, indicating that dynamic quenching can be the dominant mechanism of the observed fluorescence quenching of the CNDs in the presence of ferric ions (Figure 8). It was further confirmed by observing that at higher temperatures, the positive deviation from Stern–Volmer linearity under one-photon and two-photon excitation is more evident, especially in the one-photon regime.

**Figure 8:**
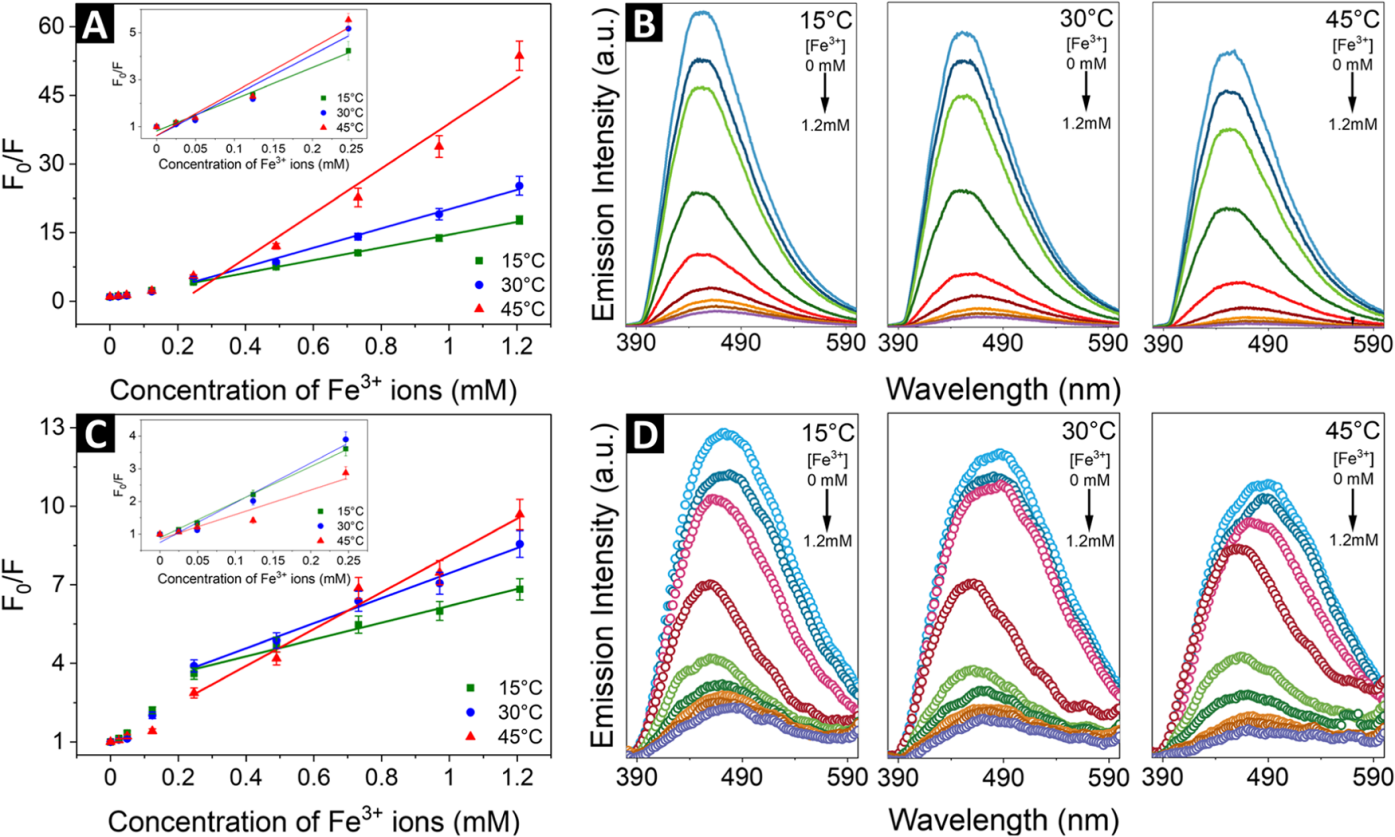
Stern–Volmer plots for CNDs in the presence of Fe^3+^ ions in different temperatures with their corresponding spectra in (A, B) one-photon (λ_exc._ = 360 nm) and (C, D) two-photon regime (λ_exc._ = 720 nm).

**Table 2:** Values of the Slope of Stern–Volmer Plot at Different Temperatures^a^

	one-photon excitation				two-photon excitation			
concentration range (mM)	0–0.25		0.25–1.2		0–0.25		0.25–1.2	
temperature	slope	*R* ^2^	slope	*R* ^2^	slope	*R* ^2^	slope	*R* ^2^
15 °C	13.50	0.984	13.91	0.996	10.89	0.994	3.21	0.981
30 °C	17.24	0.934	21.06	0.987	12.18	0.958	4.78	0.986
45 °C	18.63	0.936	49.01	0.945	7.51	0.897	7.03	0.966

a One-photon excitation wavelength is 360 nm, and a two-photon excitation wavelength is 720 nm.

Increased temperature of the system results in more frequent collisions between the quencher and the emitter. Since dynamic quenching involves collisional interactions, the likelihood of quenching events increases, resulting in greater positive deviations from the expected linear relationship in the SV plot. However, the changes of the SV plot slope at low concentrations of ferric ions (0–0.25 mM) in two-photon regime suggest a potential static quenching mechanism (see: inset of Figure 8C).

As the two-photon excitation is confined to a highly localized volume close to the focal point of the laser beam, the contribution from static quenching might stem from the spatial restriction and shifts in local molecular interactions. In the presence of higher ferric ion concentrations (>0.25 mM), both one- and two-photon excitation regimes exhibit increasing *K*_SV_ values with rising temperature, suggesting that dynamic quenching becomes a more prominent mechanism over this range of concentrations. Based on the obtained results, across both low and high concentrations of ferric ions, the fluorescence quenching in the one-photon regime is primarily attributed to dynamic quenching, involving nonradiative quenching processes, most likely charge transfer. In contrast, in the two-photon regime, the fluorescence quenching process might initially involve some contribution from static quenching, which is followed by nonradiative processes upon reaching a critical threshold of the quencher concentration.

### Cytotoxicity Studies

The CNDs exhibit a great potential for wide-ranging applications in biological systems; thus, a particularly important factor is their degree of toxicity. To evaluate whether the CNDs synthesized in this work possess a significant cytotoxicity effect on cells, two methods were utilized: the cell proliferation assay using 3-(4,5-dimethylthiazol-2yl)-5-(3-carboxymethoxyphenyl)-2-(4-sulfophenyl)-2*H*-tetrazolium (MTS) and confocal microscopic cell imaging.

The cell viability MTS assay carried out on HeLa and MCF-7 cell lines revealed that the CNDs can indeed be used in living cells: as demonstrated in Figure 9A, no significant reduction in cell viability was observed after 24 h of incubation with the CNDs at concentrations ranging from 0 to 200 μg/mL. Thus, it was concluded that the obtained nanodots are noncytotoxic, even at high concentrations.

**Figure 9:**
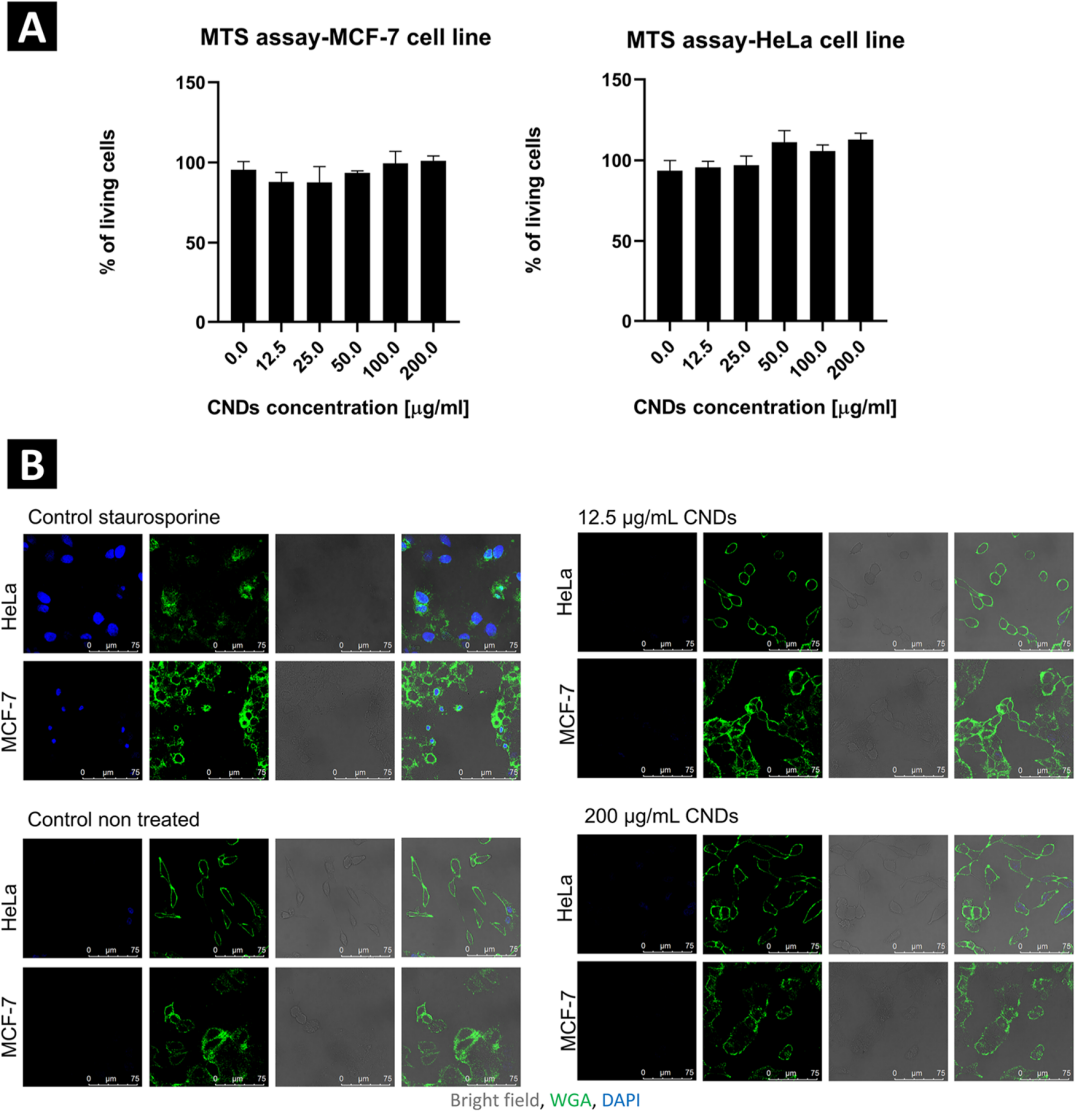
(A) MTS assay results demonstrating no significant impact of the as-synthesized CNDs on MCF-7 and HeLa cells viability; (B) confocal microscopy images presenting morphology (WGA, green) of tested cell lines incubated with two concentrations of CNDs (12.5 and 200 μg/mL), nontreated cells as negative control, and cells with STS-induced apoptosis as positive control. Data are presented as representatives from three biological replicates.

To further confirm the MTS assay results, the morphology of HeLa and MCF-7 cell lines in the presence of the CNDs was examined. Imaging of cells stained with wheat germ agglutinin (WGA, to label cell membranes, green) and 4′,6-diamidino-2-phenylindole (DAPI, which intercalates with DNA in dead cells, blue) showed that the treatment with as-synthesized CNDs did not impact cell morphology and confirmed no negative influence on the number of live, functional cells in the sample (Figure 9B). The nuclei of cells incubated with the CNDs were not stained with DAPI, suggesting the integrity of the cellular membrane and, consequently, preserved cell viability. For the staining control, apoptosis was induced in cells using staurosporine (STS), a broad spectrum protein kinase inhibitor. Nuclear staining was observed in some cells, indicating both cell death and proper DAPI staining. Additionally, the samples were stained with WGA for visual observation of cells shape. No differences were observed between untreated cells (control) and cells incubated with as-synthesized CNDs, while in the control treated with STS, the changes in the shape of cells were detected, suggesting cellular apoptosis. Incubation of HeLa and MCF-7 cells with CNDs at low (12.5 μg/mL) and high (200 μg/mL) concentrations for 24 h did not affect cell shape and its viability, demonstrating the nontoxic nature of the studied nanodots. To ensure accuracy, the viability of the cells used in our study was assessed using classical trypan blue staining, which indicated 93–95% cell viability (MCF-7, HeLa accordingly). This further confirms the integrity of the cell membrane.

## Conclusions

We investigated the behavior of colloidal, water-soluble, nontoxic CNDs as a promising fluorescent sensor for metal ions. In this study, the sensitivity and selectivity of the CNDs to Fe^3+^ ions in both the one-photon and two-photon excitation regimes was explored, revealing the potential for fluorescence quenching-based detection of these ions. To elucidate the mechanisms underlying the observed quenching, temperature-dependent fluorescence quenching experiments in both excitation regimes were conducted, which, to the best of our knowledge, is the first such demonstration for a two-photon active CNDs-based metal ions sensor. The presented findings indicate that the fluorescence quenching of the CNDs emitters primarily occurs via the dynamic quenching mechanism, although a contribution from static quenching is also observed. Moreover, a fundamental two-photon management strategy for enhancing the performance of the CNDs as versatile fluorophores under excitation in the NIR range close to the so-called “windows of biological transparency” was presented. Such an approach is crucial, as NIR wavelengths, longer than the visible light, can more readily penetrate dense regions of gases, liquids, and particulate matter with reduced scattering and absorption. Additionally, the nontoxicity of the as-synthesized CNDs was confirmed, which enables safe application of this nanomaterial in biological systems. The presented work has the potential to not only advance the understanding of the quenching mechanisms but also pave the way for the development of high-quality sensors that leverage the unique properties of CNDs for versatile optical sensing in both environmental and biomedical applications.

## Materials and Methods

### Materials

Citric acid, urea, copper­(II) chloride dihydrate, cadmium nitrate tetrahydrate, chromium­(III) nitrate nonahydrate, and iron­(III) chloride hexahydrate were purchased from Sigma-Aldrich. Aluminum chloride was purchased from Hadron Scientific. High-purity water (Milli-Q quality, resistivity <0.06 μS/cm) obtained from the Hydrolab HLP 5 UV water purification system was used throughout all of the experiments.

### CNDs Synthesis

The CNDs were synthesized using the microwave-assisted method following the protocol established by Qu et al.,^44^ with some changes. Briefly, 3 g of citric acid and 3 g of urea were added to 10 mL of high-purity water and placed on a magnetic stirrer for 30 min. Then, the solution was transferred to a Teflon-lined reaction vessel and placed in a microwave reactor (MAGNUM II, Ertec, Poland) set at 250 °C for 10 min, with a maximum of 45 bar pressure during the reaction. The dark-brown aqueous solution was then centrifuged at 8000 rpm for 20 min to collect the supernatant. The as-obtained CNDs were stored at 4 °C for further use.

### Ions Sensitivity Studies

To evaluate the sensitivity toward selected metal ions, 50 mM stock solutions of each metal ion were prepared. A total of 10 μL of a diluted CNDs aqueous solution (2 mg/mL) was introduced into 1000 μL of high-purity water. Then, different volumes of stock ion solutions were added to achieve various concentrations of metal ions. The samples were mixed, and after an incubation time of 1.5 min, the fluorescence response to the metal ions was measured.

### Morphology, Chemical Composition, and Structural Characterization

The morphology and elemental composition of the prepared CNDs was investigated with a FEI Tecnai G^2^ 20 X-TWIN transmission electron microscope, coupled with an energy-dispersive X-ray spectrometer. Sample preparation involved depositing a diluted solution of the CNDs onto copper grids, followed by solvent evaporation. The powder X-ray diffraction (PXRD) data were collected using a Proto AXRD Benchtop powder diffractometer with a Cu Kα anode (λ = 0.1542 nm) operating at 30 kV and 20 mA equipped with a Dectris Mythen2 R 1D hybrid photon counting detector and Soller slits.

### Optical Characterization

The absorption spectra of the synthesized colloidal CNDs were measured by using a JASCO V-730 UV–vis spectrophotometer, while the one-photon excited emission spectra were recorded by using a HORIBA FluoroMax-4 spectrofluorometer. The photoluminescence QY values for the CNDs were calculated based on the comparative method, using Coumarin 153 in EtOH (QY = 0.544 ^45^) and fluorescein in 0.1 M NaOH solution (QY = 0.92 ^46^) as reference standards. To determine the nonlinear optical (NLO) characteristics, a laser system capable of delivering ∼55 fs laser pulses at the repetition rate of 1 kHz in the range of wavelengths from 600 to 1500 nm was used, comprising a Coherent Astrella ultrafast regenerative amplifier operating as the 800 nm pump, followed by a TOPAS-PRIME optical parametric amplifier (manufactured by Light Conversion). The calculated intensity at the focal point was roughly 100 GW/cm^2^, with pulse energies around 5 μJ, corresponding to an average power of approximately 5 mW. The two-photon excited emission spectra were acquired using an Ocean Optics Insight Flame CCD fiber-optic spectrometer with dedicated software. The quantitative determination of two-photon absorption cross sections of the CNDs was based on the two-photon excited emission method (TPEE) as described by Makarov et al.,^30^ using a Coumarin 153 solution as a reference dye (see details in Supporting Information). For the two-photon excited luminescence ion sensing experiment, the beam of the femtosecond laser system, tuned to the optimal wavelength (corresponding to the maximum of the two-photon absorption cross-section, σ_2_), was focused by a lens onto a quartz cuvette (path length of 1 cm) containing colloidal samples. The emission was collected perpendicularly to the excitation laser beam by a pair of lenses and an optical fiber connected to the Ocean Optics Insight QE-Pro-FL spectrometer. Cut-off short-pass filters with the wavelength of 750 or 600 nm (depending on the excitation wavelength range) were placed before the spectrometer to minimize the signal from scattering of the excitation laser beam. To prevent the generation of a white-light continuum, the laser power was attenuated using neutral-density filters. The temperature dependence measurements were performed in a Peltier-controlled cuvette holder (qX3, Quantum Northwest) with associated TC 1 Temperature Controller (Quantum Northwest). The luminescence kinetics of the synthesized CNDs as well as lifetime reduction upon addition of various amounts of 50 mM stock solution of ferric ions were measured with the use of a time-correlated single-photon counting (TCSPC) self-constructed setup based on Becker & Hickl hardware, consisting of a data acquisition module (SPC-130-EM) and a hybrid PMT detector (HPM-100-06) mounted to a Priceton Instruments spectrograph (Acton SpectraPro-2300i) under excitation with a 375 nm picosecond laser diode (BDL-375-SMC). The dedicated Becker & Hickl SPCImage software was used for the τ value calculation. The double-exponential model was used for fitting, giving the results of τ_1_ and τ_2_ values with the percentage share and final τ_m_ values. Time-resolved photoluminescence spectroscopy (TRPL) with femtosecond (λ_exc._ = 720 nm) pulse excitation was performed with the setup composed of a regenerative amplifier laser system delivering 100 fs pulses with an energy of 1 mJ and 1 kHz repetition rate, consisting of a Coherent Libra-S all-in-one ultrafast oscillator, a Coherent OPerA-Solo optical parametric amplifier, and a Hamamatsu C5677 streak camera with 14 ps resolution.

### Cell Proliferation Assay

The cell proliferation assay was performed using HeLa and MCF-7 cell lines. Cells were seeded in the 96-well plates (Clear Flat Bottom, Coring, 3596) at a density of 7000 cells/well in DMEM (Gibco, 21063-029) supplemented with 10% FBS (Gibco, A5256801), 1% penicillin/streptomycin (Sigma-Aldrich, P4333), and 2 mM l-glutamine (Sigma-Aldrich, G7513). After 24 h of incubation, the cells were washed with DPBS (Gibco, 14190-144) and fresh medium containing different concentrations (0–200 μg/mL) of CNDs. Twenty-4 h later, 3-(4,5-dimethylthiazol-2yl)-5-(3-carboxymethoxyphenyl)-2-(4-sulfophenyl)-2H-tetrazolium (MTS) reagent was added according to the manufacturer’s protocol. The incubations were carried out at 37 °C with 5% CO_2_. Absorbance at 490 nm was measured using a spectrofluorometer (Molecular Devices). The results were analyzed with SoftMax and GraphPad Prism software. Data are presented as mean values with a standard deviation SD from three biological replicates.

### Confocal Microscopy Imaging

HeLa and MCF-7 cells (1 × 10^5^ cells/mL) were incubated on coverslips in DMEM (Gibco, 21063-029) at 37 °C with 5% CO_2_ for 24 h. Cells were then washed with DPBS (Gibco, 14190-144). Next, the control sample was treated with 1 μM of staurosporine (STS, AmBeed, A256460) and incubated for 6 h at 37 °C with 5% CO_2_, while other samples were incubated with or without CNDs at concentrations of 12.5 and 200 μg/mL for 24 h. After this time, the medium was aspirated, cells were washed with DPBS, and samples with cells were stained with anti-WGA (conjugated with AF 488) for 10 min at 37 °C with 5% CO_2_, followed by incubation with 1 μg/mL of DAPI (Invitrogen, D1306) for additional 5 min. Cells were then fixed with 4% paraformaldehyde for 15 min at room temperature. After aspirating the solution, coverslips were mounted with Fluoromount G (Invitrogen, 00-4958-02). The slides were imaged using a Leica TCS SP8 confocal microscope with excitation provided by a 488 nm solid-state laser for WGA (green) and a 405 nm diode laser for DAPI (blue).

## Supplementary Material



## Notes

PXRD data for the as-synthesized CNDs; excitation-dependent emission spectra of CNDs; fluorescence quenching effects of CNDs in the presence of different metal ions; luminescence lifetimes under one-photon and two-photon excitation of CNDs upon addition of ferric ions solution; determination of two-photon absorption cross sections (PDF)
