# Supplementary material for: Efficient Quenching of Two-Photon Absorption Induced Photoluminescence in Carbon Nanodots for Fe3+ Ion Detection
Source: ACS Omega. 2025 Jun 26;10(26):28020–31. doi: 10.1021/acsomega.5c01915 (PMC12242630; doi:10.1021/acsomega.5c01915)
Supplement: Supplementary file 1 [file ao5c01915_si_001.pdf]

Efficient quenching of two-photon absorption induced photoluminescence in carbon nanodots for Fe^{3+} ions detection

Agnieszka Siomra[a], Dominika Wawrzyńczyk[a], Bartłomiej Cichy[b], Magdalena Wądrzyk[c], Paulina Kasperkiewicz[c], Marek Samoć[a], Marcin Nyk[a]*

[a] Institute of Advanced Materials, Faculty of Chemistry, Wroclaw University of Science and Technology, Wybrzeze Wyspianskiego 27, 50-370 Wroclaw, Poland.

[b] Polish Academy of Sciences, Institute of Low Temperature and Structure Research, Okolna 2, 50-422 Wroclaw, Poland

[c] Department of Chemical Biology and Bioimaging, Wroclaw University of Science and Technology, Na Grobli 15, 50-421 Wroclaw, Poland

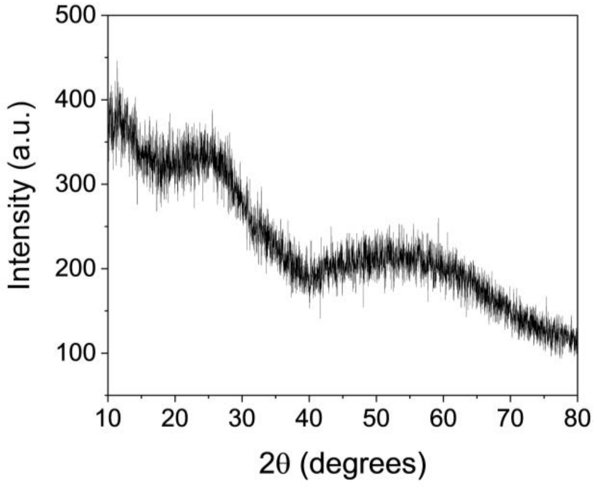

Figure S1. Powder X-ray diffractogram for the as-synthesized CNDs.

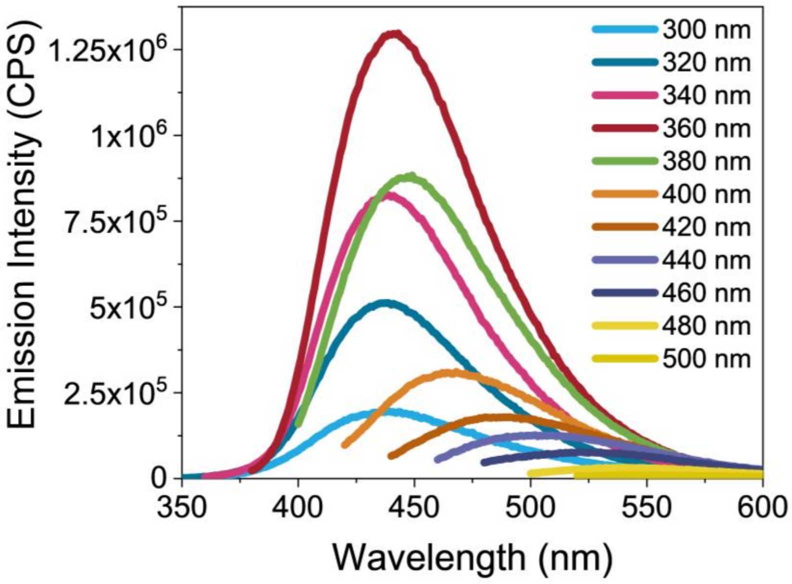

Figure S2. Excitation dependent emission spectra of the as-synthesized CNDs.

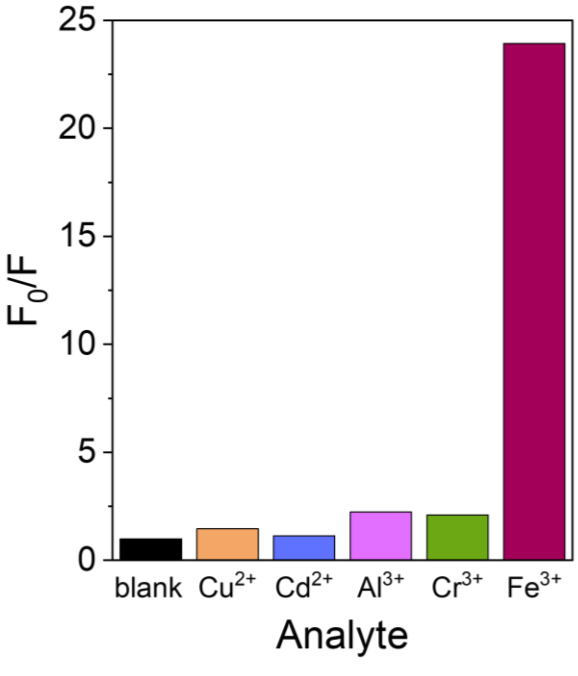

Figure S3. The fluorescence quenching effects of CNDs in the presence of different metal ions at the concentration of 2 mM.

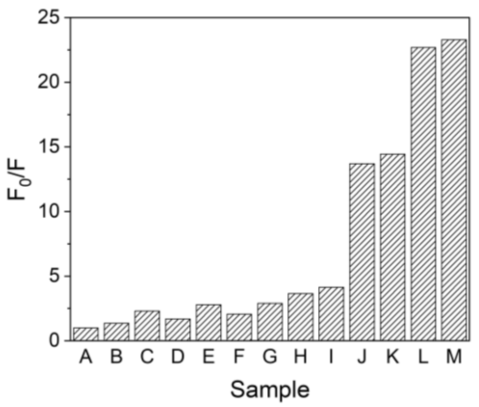

Sample	Added volume of Cu^{2+} solution	Added volume of Cd^{2+} solution	Added volume of Al^{3+} solution	Added volume of Cr^{3+} solution	Added volume of Fe^{3+} solution
	µL	µL	µL	µL	µL
A	0	0	0	0	0
B	10	10	0	0	0
C	0	0	10	10	0
D	30	30	0	0	0
E	0	0	30	30	0
F	50	50	0	0	0
G	0	0	50	50	0
H	10	10	0	0	5
I	0	0	10	10	5
J	30	30	0	0	25
K	0	0	30	30	25
L	50	50	0	0	35
M	50	50	50	50	35

Figure S4. Changes of the F_0/F parameter of the CNDs upon addition of various volumes of 50 mM Fe^{3+} ions and interferent (Cd^{2+}, Cu^{2+}, Al^{3+}, Cr^{3+}) ions solutions.

Table S1. Limits of detection (LoD) and limits of quantification (LoQ) reported for existing fluorescent probes for Fe^{3+} detection

Material	One-photon regime		Two-photon regime		Reference
	LoD	LoQ	LoD	LoQ	
**N-CNDs conjugated with fluorescein isothiocyanate**	1.56 nM	~4.7 nM*	2.21 nM	~6.6 nM*	1
**N-CQDs (biomass derived)**	0.75 µM	~2.2 µM*	-	-	2
**CQDs (biomass derived)**	2.01 µM	~6.0 µM*	-	-	3
**Graphene QDs (biomass derived)**	2.5 ± 0.3 µM	7.6 ± 0.9 µM	-	-	4
**Polyamine-functionalized CNDs**	66.7 nM	~200 nM*	-	-	5
**N-CQDs**	55.7 µM (water)<br>40.2 µM (FBS)	~167 µM<br>~120 µM*	-	-	6
**1,8-naphthalimide-based fluorescent probe**	65.2 nM	~195 nM*	-	-	7
**Rhodamine-based fluorescent probe**	5.2 µM	~15.6 µM*	-	-	8
**Label free CNDs**	53 µM	160 µM	80 µM	256 µM	This work

Table S2. One-photon excited luminescence lifetimes (short and long components, together with mean values) for CNDs upon sequential addition of 50 mM ferric ions solution.

**Amount of Fe^{3+} ions solution added [uL]**	**τ_1 [ns]**	**τ_2 [ns]**	**τ_m [ns]**
**0**	2.99 (26%)	8.81 (74%)	7.31
**5**	4.05 (40%)	9.69 (60%)	7.44
**10**	4.13 (42%)	9.74 (58%)	7.42
**15**	2.38 (24%)	8.47 (76%)	7.03
**20**	3.15 (30%)	8.94 (70%)	7.21
**40**	1.83 (27%)	9.44 (73%)	7.38
**60**	3.62 (37%)	8.78 (63%)	6.88
**80**	4.12 (61%)	9.87 (39%)	6.35
**100**	1.78 (31%)	7.11 (69%)	5.45
**120**	1.77 (38%)	6.86 (62%)	4.91
**140**	2.15 (37%)	6.50 (63%)	4.90
**160**	1.78 (37%)	6.33 (63%)	4.63
**180**	1.64 (34%)	5.80 (66%)	4.39
**200**	1.43 (39%)	5.69 (61%)	4.03

Table S3. Calculated luminescence lifetime values upon two-photon excitation (λ_{exc} = 720 nm) for CNDs upon sequential addition of 50 mM ferric ions solution.

**Amount of Fe^{3+} ions solution added [uL]**	**τ_1 [ns]**
**0**	6.59
**5**	2.01
**10**	1.32
**15**	0.89
**20**	0.50
**40**	0.29

Determination of two-photon absorption cross sections

The two-photon absorption cross sections (σ_2) were determined by TPEE method described by Makarov *et al.*[9] and calculated using the equation:

$$\sigma_2 = \sigma_{2,ref} \frac{QY_{ref} \cdot c_{ref}}{QY \cdot c} \cdot \frac{P_{ref}^2}{P^2} \cdot \frac{I}{I_{ref}} \cdot K$$

with the correction factor for the differences in the refractive index of sample and reference solvents described as:

$$K = \frac{n^2}{n_{ref}^2}$$

where:

σ_2, $\sigma_{2,ref}$ – two-photon absorption cross section of the sample and the reference

QY, QY_{ref} – quantum yield of the sample and the reference

c, c_{ref} – concentration of the sample and the reference

P, P_{ref} – laser beam power for the sample and the reference

I, I_{ref} – integrated emission intensities of the sample and the reference

n, n_{ref} – refractive index of the sample and the reference solvent

The excitation wavelength-dependent values of $\sigma_{2,ref}$ for coumarin 153 used in the calculations were obtained from the study by Makarov *et al*[9]. The QY of the CNDs was determined using the comparative method, with coumarin 153 in CCl_4 (QY = 0.544[10]) and fluorescein in 0.1M NaOH solution (QY=0.92[11]) as reference standards. Two-photon excited emission measurements for both the sample and the reference dye were performed under identical experimental conditions, with the calculated excitation intensity at the focal point of roughly 100 GW/cm^2 and pulse energies around 5 μJ.

References:

(1) Lesani, P.; Singh, G.; Viray, C. M.; Ramaswamy, Y.; Zhu, D. M.; Kingshott, P.; Lu, Z.; Zreiqat, H. Two-Photon Dual-Emissive Carbon Dot-Based probe: Deep-Tissue imaging and ultrasensitive sensing of intracellular ferric ions. *ACS Applied Materials & Interfaces* **2020**, 12 (16), 18395–18406. DOI: 10.1021/acsami.0c05217.

(2) Qi, H.; Teng, M.; Liu, M.; Liu, S.; Li, J.; Yu, H.; Teng, C.; Huang, Z.; Liu, H.; Shao, Q.; Umar, A.; Ding, T.; Gao, Q.; Guo, Z. Biomass-derived nitrogen-doped carbon quantum dots: highly selective fluorescent probe for detecting Fe^{3+} ions and tetracyclines. *Journal of Colloid and Interface Science* **2018**, *539*, 332–341. DOI: 10.1016/j.jcis.2018.12.047.

(3) Nagaraj, M.; Ramalingam, S.; Murugan, C.; Aldawood, S.; Jin, J.-O.; Choi, I.; Kim, M. Detection of Fe^{3+} ions in aqueous environment using fluorescent carbon quantum dots synthesized from endosperm of Borassus flabellifer. *Environmental Research* **2022**, *212*, 113273. DOI: 10.1016/j.envres.2022.113273.

(4) Abbas, A.; Tabish, T. A.; Bull, S. J.; Lim, T. M.; Phan, A. N. High yield synthesis of graphene quantum dots from biomass waste as a highly selective probe for Fe^{3+} sensing. *Scientific Reports* **2020**, *10* (1), 21262 . DOI: 10.1038/s41598-020-78070-2.

(5) Zhao, L.; Geng, F.; Di, F.; Guo, L.-H.; Wan, B.; Yang, Y.; Zhang, H.; Sun, G. Polyamine-functionalized carbon nanodots: a novel chemiluminescence probe for selective detection of iron(III) ions. *RSC Advances* **2014**, 4 (86), 45768–45771. DOI: 10.1039/c4ra08071h.

(6) Van Huan, P. Multicolor nitrogen-doped carbon quantum dots and its application in the detection of Fe^{3+} ion. *Luminescence* **2024**, 39 (8), e4852. DOI: 10.1002/bio.4852.

(7) Li, X.; Qin, W. A novel dual-capability naphthalimide-based fluorescent probe for Fe^{3+} ion detection and lysosomal tracking in living cells. *RSC Advances* **2022**, 12 (37), 24252–24259. DOI: 10.1039/d2ra03688f.

(8) Zhang, M.; Shen, C.; Jia, T.; Qiu, J.; Zhu, H.; Gao, Y. One-step synthesis of rhodamine-based Fe^{3+} fluorescent probes via Mannich reaction and its application in living cell imaging. *Spectrochimica Acta Part a Molecular and Biomolecular Spectroscopy* **2020**, *231*, 118105. DOI: 10.1016/j.saa.2020.118105.

(9) Makarov, N. S.; Drobizhev, M.; Rebane, A. Two-photon absorption standards in the 550–1600 nm excitation wavelength range. *Optics Express* **2008**, 16 (6), 4029-4047. DOI: 10.1364/OE.16.004029

(10) Rurack, K.; Spieles, M. Fluorescence Quantum Yields of a Series of Red and Near-Infrared Dyes Emitting at 600−1000 nm. *Analytical Chemistry* **2011**, 83 (4), 1232-1242. DOI: 10.1021/ac101329h.

(11) Brouwer, A. M. Standards for photoluminescence quantum yield measurements in solution (IUPAC Technical Report). *Pure and Applied Chemistry* **2011**, 83 (12), 2213-2228. DOI: 10.1351/PAC-REP-10-09-31.
